# Evaluation of Expressive Arts Therapy on the Resilience of University Students in COVID-19: A Network Analysis Approach

**DOI:** 10.3390/ijerph19137658

**Published:** 2022-06-23

**Authors:** Yue Li, Jun Peng

**Affiliations:** 1 Division of Arts, Shenzhen University, Shenzhen 518060, China; lee@szu.edu.cn; 2 School of Education, Research Institute of Macau Education Development, City University of Macau, Macau 999078, China

**Keywords:** university students, resilience, expressive arts therapy, network analysis, COVID-19

## Abstract

As an alternative to traditional verbal counselling, expressive arts therapy has been shown to be an effective method of mental health care, particularly when dealing with stressful public interactions, such as those associated with COVID-19. However, few studies have been conducted to determine the efficacy of expressive arts therapy on the resilience of psychologically exposed university students during COVID-19. Furthermore, since network analysis appears to be a popular approach in psychological research, it has not been used in recent intervention studies for resilience. As a result, the current study utilized a network analysis approach to determine the efficacy of expressive arts therapy on the resilience of university students during the COVID-19 pandemic. A total of 263 students in a comprehensive university in China were selected for the therapy group between March and November 2021. In a pre-post design, students’ resilience was assessed using the Resiliency Scale for University Students (RSUS). The extended Bayesian information criteria (EBIC) and graphical LASSO were used to estimate and define paired resilience networks, and the strength, betweenness, and closeness indices were utilized to determine the centrality of the six facets of resilience. Additionally, we verified the stability and accuracy. It was discovered that significant differences appeared between the paired networks before and after expressive arts therapy. Facets of self-efficacy, self-acceptance and problem-solving in resilience were notably improved after the therapy, with the variable of emotional stability sustained at the mean level. Meanwhile, the network analysis has highlighted the central variable of self-efficacy in the pre-intervention and support from friends in the post-intervention. The connectivity among the components of problem solving, support from friends, and support from family was enhanced, with support from friends playing the role of hub nod in the following network. By utilizing a network analytic approach, expressive arts therapy can be more targeted in intervening in resilience mechanisms. As a proxy for efficacious problem-solving, intervention should be calibrated to the cultivation of social support networks, especially in the support from friends.

## 1. Introduction

### 1.1. Resilience of University Students

An individuals’ ability to cope with stress is influenced by their extent of resilience, which can drive them to cope more effectively when faced with stressful circumstances [1]. In other words, resilience refers to the capabilities to adapt well and promote positive change in the face of adversity [2], stress [3], trauma [4], and even a direct threat [4]. Accordingly, resilience, as a prominent indicator of mental health under stressful circumstances, deserves extensive attention.

A wide range of scholars have increasingly observed the protective role of resilience in the psychological state of university students in the epidemic. As repeated measures of collective quarantine altered previous patterns of social interactions between people, university students are particularly more vulnerable than the general population, given their lack of life experience, limited psychological tolerance, lack of stability, undeveloped cognitive capacities, defense mechanisms, and frequent inability to deal with unexpected events [5]. The continuous disconnection from social life [6], insufficient social support [7], combined with the poor economic environment [8], exposed the college student population to psychological problems, such as anxiety [9], depression [10], sleep problems [11], and PTSD symptoms [12]. Fortunately, resilience has been proved to exert a positive influence on psychological well-being in a vast array of university students, from China to Spain [13,14,15]. In this sense, enhancing the resilience of university students should be the priority to cope with the negative events and emotions in the ongoing environment of COVID-19.

### 1.2. Intervention in Resilience during the COVID-19 Pandemic

Given that psychological intervention in resilience is highly recommended in the pandemic-related public health emergencies [16,17], a small number of studies of intervention specific resilience have been observed. Remote psychological counseling is the most used means of psychological intervention in the school context. In a sample of 160 Chinese adolescents, an intervention of psychological counseling combined with outdoor exercise proved to have an effect on resilience during the early stage of the COVID-19 pandemic [18]. Combined with a semi structured interview and questionnaire, an Italian study concluded that online counseling intervention inclusive of telephone listening services, paired with psychoeducational workshops in quarantine, could improve the resilience of university students [19]. Researchers in Indonesia developed a mobile-based counseling application and confirmed its facilitating efficacy, compared to the treatment-as-usual, in the promotion of resilience among students with academic problems during the pandemic [18].

A recent Chinese study, which engaged in COVID-19 related coping strategies and anxiety among university students, advocated that school counselors should manipulate interventions other than verbal techniques to enhance the social support and emotional coping of students [20]. Therefore, in addition to verbal counseling, some scholars began to adopt behavioral intervention. An eight-week multifaceted mindfulness-based self-care program targeted at 123 senior medical students in Chile was implemented during April to May 2020 and reported increased resilience of the students and showed the effectiveness of intervention in self-awareness and self-regulation [21]. A recent study in a Chinese sample performed an experiment of mindfulness training on 90 students as well, exploring the developmental trajectories of the impact of mindfulness training on resilience, with the finding of increased individual resilience and emotional intelligence [22]. The 6-year (2012–2017) Youth-Led Resilience Promotion program that was originally conducted to equip youth leaders with resilience and prosocial behaviors in the aftermath of a school shooting could also be adopted to traumatic events, such as the COVID-19 pandemic, to boost psychological recovery [23]. Although various means of intervention have been conducted, only limited efforts have been carried out to clarify the influence of arts therapy on the resilience of university students in COVID-19 related stress.

### 1.3. Rationale for Intervention of Expressive Arts Therapy in Resilience

Psychological issues such as anxiety, depression, post-traumatic stress disorder (PTSD), schizophrenia, and substance misuse disorders can be treated through arts therapy. Visual arts [24], music [25], dance [26], drama [27], and other expressive media are used by arts therapists to help individuals grow and develop. It should be highlighted that arts therapists may deal with clients one-on-one or in groups with problems and the targeted subjects include people in all ages, from children to adults. As a useful technique, arts therapy has been utilized for many years in hospitals and psychiatric wards [28,29], but now it is also being used in the community as well as in schools [30] and colleges [31,32,33]. Hence, the main purpose of this study is to take advantage of expressive arts therapy and continue exploring the efficacy in enhancing resilience concerning university students. 

Expressive arts therapy is one creative avenue to meet the process-oriented and structure-oriented desire in resilience intervention. From one side, the aforementioned empirical studies elucidated that resilience could be trained and bolstered, which verified the process-context model of resilience that resilience was more precisely viewed as processes rather than a stable trait [34,35]. Expressive arts therapy refers to the uniquely designed practice that applies imagination, rituals, and the creative process to address psychological issues by melding with a wide range of arts modalities, such as painting, drawing, sculpture, dance, movement, music, drama, poetry, or prose [36]. Notably, it has recognized the significance of arts for being the change agent [37]. Based on the theoretical foundation of social action, expressive arts therapy is rooted in a social action model that inclusively encourages collaboration, community building, and social change creation [37]. The unique powerful therapeutic contribution of creating change echoes the changing process of resilience.

From the other side, the structured feature of resilience can be captured by the definition as follows: it is not possible to classify resilience as a single trait of a person, instead, it is a combination of internal resilient characteristics and environmental-psychological factors that interact to produce resilience [38]. Special attention on the constructs of resilience in the COVID-19 pandemic situation has already been raised. A cross-sectional study of 5530 university students, which found the moderating power of resilience from distress to COVID-19 prevention behaviors, encouraged further research to examine the differing constructs of resilience [5]. Based on the protective model of resilience theory, promotive factors in resilience were found to work together to facilitate positive adaption [39]. With the emphasis of communicative resilience theory, seven triggers in constituting resilience were explored in a US COVID-19 related study of university students [40].

With the multidisciplinary vision, various curative factors interplay to facilitate resilience in the therapeutic mechanism of expressive arts therapy, among which six factors in the construct of resilience are worth investigating. First, the effectiveness of expressive arts therapy in self-efficacy was identified. A 4-day pilot short-term residential program entitled “RESTART” adopted expressive arts therapy on clients with addiction, restoring and facilitating the self-efficacy of the participants [41]. Secondly, by means of the non-verbal artistic expression, self-acceptance was strengthened through the reconstruction of the primary sense of the self [42]. Thirdly, in terms of emotional stability, the affirmative effect has been proven by the findings of Korean studies in college students using integrated arts therapy [43]. Fourthly, the expressive arts groups provided opportunities to develop positive coping strategies, which reinforces the problem-solving competence [31]. Fifthly, with the core value of collaboration, expressive arts therapy built communities and enhanced cohesion through support from friends. A study of student-to-student programs found that the peer support networks and the school experience were consolidated by group therapy among first-year college students [44]. Finally, family resilience has been boosted by creative arts therapies. The efficacy of art therapy, dance/movement therapy, and music therapy in encouraging support from families was realized by discovering strengths, inspiring connections, and improving understanding of family relationships [45].

In the context of the COVID-19 crisis, diverse patterns of expressive arts therapy in promoting resilience have been documented, such as art therapy, dance therapy, music therapy, and drama therapy. The benefits of visual esthetic exposure on the activation of the reward systems, which in turn stimulate mental resilience, have been reported within the widespread pandemic context [46]. A qualitative study revealed the supportive impact of online music programs of group-singing on the adaptability and resilience of people with dementia and their family caregivers [47]. An intervention of dance therapy based on the Satir model investigated 62 adolescents in China and found that the experiment group reaped more benefits of psychological resilience than the control group, indicating the feasibility of the group dance therapy approach [48]. A pilot workshop conducted in a drama education project in Hong Kong confirmed the effectiveness of the integrated implication of play, process drama and integrated arts education in elevating the resilience of teachers during the pandemic [49].

Nevertheless, there is a dearth of empirical study addressing the resilience intervention by use of integrated expressive arts therapy. Hence, previous studies have left much room for further investigation. First, a handful of research concerning arts therapy have delved into the building of resilience, but no studies regarding integrated expressive arts therapy intervention have been conducted for resilience in the context of COVID-19. Second, most of the studies related to resilience intervention by means of arts therapy were implemented in clinically diagnosed patients’ samples, rather than university students, while exploring optimal interventions to empower college students is crucial in the pandemic. Third, although the efficacy of various curative factors in resilience has been explored respectively, little is known about the interplay and the overall structure of the components of resilience through the intervention of expressive arts therapy. Since arts interventions have faced challenges of scarce scientific rating instruments [50], novel assessment measures of the effectiveness need to be identified and implemented. However, network analysis, as an innovative approach to illustrate the interplay of psychological elements based on graph theory, displayed the causal systems of psychological phenomena consisting of mutually influenced components [51]. On the one hand, this bottom-up approach does not demand strong theoretical assumptions or predictions in the literature about the interrelations of variables in the modelling [52]. On the other hand, by identifying the most central element and revealing the unknown network connections in the network [52], the network unveils a specific hypothesis system that is composed of causal links (i.e., edges) among the components (i.e., nodes) [53]. In this way, the network analysis approach can be applied to investigate the causal interrelationship of various constructs among resilience, preventing the limitation of cross-sectional survey methodology.

### 1.4. The Present Study

Based on the literature review above, we sought to address the research gap by conducting an intervention adeptly designed for resilience by means of expressive arts therapy tailored to university students in the COVID-19 context. Meanwhile, we adopted a network analysis approach to shed light on the evaluation of the art therapy intervention in a pre-post design. We hypothesized that (1) the six components in the resilience (i.e., self-efficacy, self-acceptance, emotional stability, problem-solving, support from friends and support from family) would be enhanced significantly, and (2) the network of the constructs among resilience would be changed after the expressive arts therapy. Hence, the present study took a preliminary step in mapping the relationship between the components within resilience through a network analysis approach and depicting more comprehensive and dynamic understanding of the influence of expressive arts therapy on resilience. The findings would underpin the implication of expressive arts therapy to promote the mental health of university students in the COVID-19 pandemic.

## 2. Methods

### 2.1. Participants

The students were first notified of the propaganda about the psychological growth training workshop, and students with interests were surveyed in interviews with open-ended questions about challenges due to the COVID-19 related circumstances. Under the approval of the Institutional Review Board, this study recruited a sample of participants with the following criteria: (a) the participants must be enrolled in a comprehensive university in China, (b) they must be a freshman at least 17 years old, (c) they must have experienced at least one challenge in response to the COVID-19 situational context. The challenge referred specifically to stress experienced owing to the negative events during the epidemic, including academic problems, difficulties in intimacy, adjustment difficulties, poor social networks and external threats related to the pandemic. Accordingly, 274 university students from freshmen classes who were self-aware of their own emotions of distress and persistent sadness were given the informed consent and considered to be recruited. Since 11 dropped out of the study for personal reasons, 263 students (mean age = 18.86 ± 0.97 years; females, 221) finally received intervention as the treatment group between March and November 2021.

### 2.2. Measures

#### Resiliency Scale for University Students (RSUS)

The Resiliency Scale for University Students (RSUS) [54] was used to assess the tendencies in coping with stress in the context of COVID-19. A total of 31 items in the RSUS were rated on a 5-point Likert scale ranging from 1 (“strongly disagree”) to 5 (“strongly agree”). There were six dimensions in the RSUS, which were as follows: self-efficacy (SE; item 1–5), self-acceptance (SA; item 6–8), emotional stability (ES; item 9–15), problem-solving (PS; item 16–18), support from friends (SFRI; item 19–26) and support from family (SFAM; item 27–31). The scale SE evaluated the degree to which individuals affirmed their self-competence, and the items were listed such as “I am confident in my abilities” and “if I try, I can do things as well or better than anyone else”. The scale SA referred to the extent to which individuals affirmed their self-worth and accepted the reality of their selves, and the examples of items were “I still feel pretty good about myself” and “thinking about my strengths and weaknesses, I feel relatively satisfied with myself”. Items such as “I often get depressed for no apparent reason” and “my mood fluctuates from time to time” represented the scale ES, which valued the personality traits that made an individual emotionally stable and mature, including the speed of emotional change and emotional susceptibility. The scale PS measured the tendency of individuals to solve problems through their own strategies of planning, execution, and time management when faced with stressful situations, with items listed as “when things get tough, I often make a plan and stick to it”. The scale SFRI indicated perceptions of support from friends, including objective, emotional and cognitive and strategic support, with items such as as “I can find some friends to share my joys and sorrows with me” and “when I am in trouble, I often ask my friends for help”. The scale SFAM examined the perceived support from family members, both objective and emotional, with items such as “I often get emotional support from my family” and “I often get a lot of support from my family when I’m having a hard time”. Higher mean scores indicated greater availability of resilience, with some items in the ES subscale being reversely coded. Reliability was previously found to be satisfying, as reflected in good Cronbach’s alpha coefficients (SE, 0.73; SA, 0.80; ES, 0.84; PS, 0.64; SFRI, 0.79; SFAM 0.69) [54]. Retest reliability in the present study from pre to post was 0.47, 0.40, 0.44, 0.35, 0.33, and 0.37 among the two waves.

### 2.3. Procedures

Previous evidence in longitudinal studies in samples of Chinese college students has proved that there were no significant changes in stress, anxiety, and depression levels, and the reduction in PTSD symptoms was not clinically significant either [55]. More importantly, there was a statistically significant increment in psychological burden from the COVID-19 outbreak period to the remission period for Chinese college students [56]. Therefore, it is reasonable to suppose that Chinese college students are experiencing long-term psychological pressure under the imposition of countrywide confinement measures, and their initial resiliency was not sufficient to soothe the mental stress. In this sense, the interventions of resilience by external therapy were necessary, and the transience of the pandemic-related stress can be controlled by the sustained epidemic environment.

Participants were recruited in a sample of freshmen in a comprehensive university in China. A total of 263 participants were separated into 13 groups in this project, with approximately 20 students each group. A pre-post design with network analysis approach was implemented. Before and after the targeted expressive arts therapy, students’ resilience was measured using the Resiliency Scale for University Students (RSUS). The participants completed the assessment at the baseline (wave 1) and at the end of the intervention session (wave 2).

This workshop was guided by a psychological counselor with three years of professional training in arts therapy at college. The program aimed at providing supportive interventions to enhance the resilience in psychosocial coping under the COVID-19 pandemic context. To be specific, the workshop applied diversified patterns of expressive arts therapy to spur the students’ perceptions of self-efficacy, self-acceptance, emotional stability, problem-solving, support from friends and support from family. A four-session curriculum that integrated expressive arts therapy, including forms of music, dance, art, and drama, were designed specifically addressing the students’ psychological stress. The intervention was conducted once a week with 90 min per session. The therapist implemented the group protocol, asked the participants to sign the group ethical commitment, and conducted the intervention according to the following guidelines.

#### 2.3.1. Session 1

The purpose of the first session is to build a group and release tension through music therapy. It is of great importance to create a safe space in the first session. Therefore, in the early half of the session, the group leader builds rapport by introducing and welcoming the group members, exploring the group consensus, and addressing the rules for engagement and respect. In the latter half of the session, toning through the natural voice of vowel sounds and humming in group work is administrated, which is an effective technique of music therapy targeted at non-musician populations in the community [57]. In the alternate process of toning and meditation, participants are supposed to regulate breathing, relax the body, and release stress.

#### 2.3.2. Session 2

The goal of the second session is to build a connection with the self and with external resources through dance/movement therapy. According to the composite model of DMT for resilience-building, three modules are structured [58]. The theme of turning into the body is designed to increase body awareness and examine the body image through kinesthetic imagery; the theme of breath and flow aims at connecting one’s body and feelings; the theme of making connections is supposed to enhance cohesion and peer relationships through mirroring interactions. The implications of the group activities are supervised under the rationale of the eight healing processes that Claire developed (i.e., synchrony, expression, rhythm, vitalization, integration, cohesion, education, symbolism) [59]. After each module, participants are required to write down or confer within the group the reflections on the body, emotion, and the thought.

#### 2.3.3. Session 3

The third session focuses on expressing personal perception and increasing emotional awareness through art therapy. The students are supposed to experience and express their emotions on their stressful events through exploratory artistic work. The session is structured into three modules. First, in order to establish confidence, the participants are encouraged to explore their recourses by drawing a safe landscape and modelling a figure in clay. Second, the students are invited to express the negative emotions using artistic modalities, such as paint, clay, crayons, and stacks of magazines. Third, a supporting and comforting letter is written to the clay figure by a beloved person of the figure [42]. In this way, the students are provided with unique opportunities for self-awareness of reparative emotional experience and self-exploration of personal growth.

#### 2.3.4. Session 4

The therapeutic aim of the last session is to build group socialization, foster the strengths to conquer challenges and close the group intervention by drama therapy. Inspired by the drama project to rebuild post-COVID-19 resilience in Hong Kong, the session adopts 4-process structures [49]. First, the students are assigned to an optional theme of negative events, such as academic problems, difficulties in intimacy, adjustment difficulties, poor social networks and external threats related to the pandemic. Second, the participants create the roles in the context. Third, the students select the roles. Fourth, they improvise the play with the following thoughts: (1) I can fight the challenge; (2) what I have learned from the stress; (3) days with the stress; (4) how can I build my strengths and toughness to face the stress. By means of creative role-play, the students develop coping competence with stress and build a social microcosm of supportive dynamics. By engaging in creative arts activities, students are prompted to reach the priority of building connections and social integration.

### 2.4. Statistical Analysis

R was used to carry out all the statistical analyses. Calculations were made on the mean, standard deviation (SD), skewness, and kurtosis of all the RSUS variables summed. In the network analysis, each variable was defined as a “node”, and the associations between these variables were represented as “edges”. The strength of the connections between the nodes was reflected by the thickness of edges when network visualization was used. The color of the edge reflected the direction in which the connections were found to be significant (e.g., green edges represented positive correlations; red edges represented negative correlations) [51]. Three primary centrality indices (i.e., betweenness, closeness, and strength) were calculated to determine which symptoms were the most important in the network and which symptoms were the least important [60,61]. The strength of a node was determined by calculating the absolute total of edge weights associated with it, which indicated the importance of a certain component. A node’s betweenness was determined by the frequency with which it appeared on all the shortest pathways between other nodes, while closeness was determined by the inverse-square sum of the distances between a node and all the other nodes in the network. Previous research has shown that assessments of closeness and betweenness were unreliable to determine nodes’ importance [62]. Hence, strength was chosen as the metric to be discussed in the current study.

Some necessary steps need to be introduced. The extended Bayesian information criteria (EBIC) [63] was first used to determine the optimal penalty coefficients to merge into the least absolute shrinkage selection operator (LASSO), which was then used to sparse the redundant information matrix. Accordingly, the matrix was used to design the network. We performed the above analysis by using the package of qgraph [64] and networktools [65].

Second, we assessed the accuracy and stability of the network model using the accuracy of edge-weights and case-dropping subset bootstrap (i.e., correlation stability coefficient, CS-C) to determine whether the results were stable (1000 iterations) [66]. The R package “bootnet” was used to perform the stability analysis [67].

Following this, a permutation test called the network comparison test (NCT) was used to see if there was a difference between the networks before and after the intervention [68]. This approach compared the sum of all edge weights between the networks to assess the network’s overall strength. The strength differences for each edge were also examined between the two networks after correcting for multiple testing (Holm–Bonferroni adjustment of *p* values). The R-package “network comparison test” [69] was used to perform the above analysis.

## 3. Results

### 3.1. Descriptive Statistics

**Figure 1:**
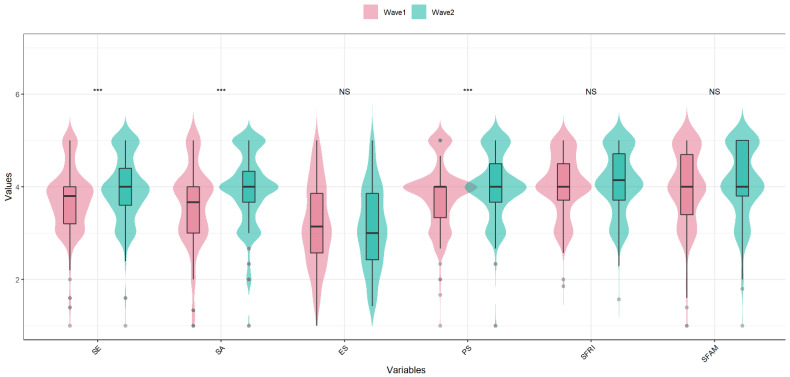
The plot of means in the paired *t*-test among wave 1 and wave 2. *** *p* < 0.001.

The mean, standard deviation, skewness, kurtosis, and polychoric correlations of all the resilience variables are reported in Tables S1 and S2 among wave 1 and wave 2, respectively. Moreover, on average, individuals tended to gain more self-efficacy, self-acceptance and problem-solving in wave 2 compared with wave 1 (*p* < 0.05), whilst emotional stability, support from friends and support from family did not change significantly (*p* > 0.05), as shown in Table 1 and Figure 1.

### 3.2. Network Estimation

**Figure 2:**
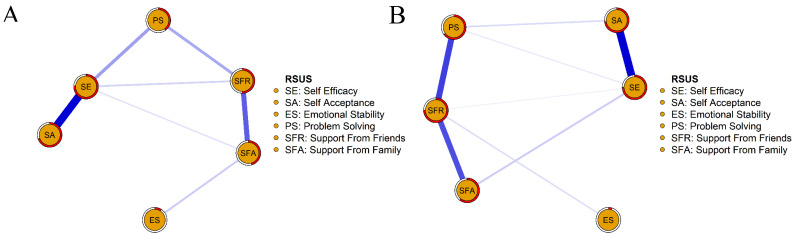
Network model for the six variables of resilience among wave 1 and wave 2. Note, positively worded items were reversed prior to the network estimation. (**A**), wave 1. (**B**), wave 2. The red circle represents the probability of a node being predicted by the surrounding connected nodes.

**Figure 3:**
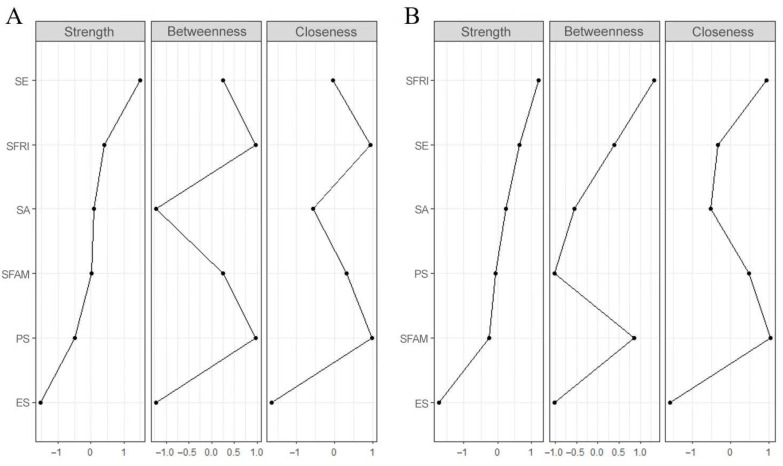
Centrality measures of all symptoms within the network. Note, the figure shows the centrality measure (i.e., strength, betweenness, and closeness) of all factors within the network (z-scores). (**A**), wave 1. (**B**), wave 2.

The networks of components in resilience among wave 1 and wave 2 are shown in Figure 2 and Figure S1. Several points are noteworthy. First, nodes of self-efficacy and self-acceptance were highly connected with the rest of the network, both among two waves. A weighted adjacency matrix was used to examine the numerical interactions between these variables (Supplementary Tables S3 and S4). Meanwhile, compared with wave 1, node PS (i.e., problem solving) and node SFR (i.e., support from friends) were enhanced with the rest of the network, and node PS (i.e., problem solving) and node SE (i.e., self-efficacy) were more loosed connected in wave 2. The centrality measurements (i.e., strength, betweenness, and closeness) of all the components in the network are depicted in Figure 3. In wave 1, variable node SE (i.e., self-efficacy) exhibited the greatest strength, whereas in wave 2, variable node SFR (i.e., support from friends) showed the greatest strength, followed by the node of self-efficacy. The node of emotional stability remained the least strong node across the two waves.

### 3.3. Network Accuracy and Stability

**Figure 4:**
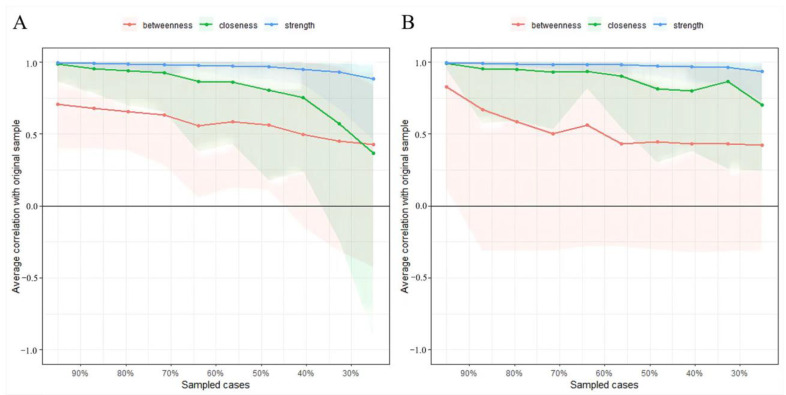
Stability of centrality indices by case dropping subset bootstrap. Note, the x-axis represents the percentage of cases of the original sample used at each step. The y-axis represents the average of correlations between the centrality indices from the original network and the centrality indices from the networks that were re-estimated after excluding increasing percentages of cases. Each line indicates the correlations among betweenness, closeness, and strength. (**A**), wave 1. (**B**), wave 2.

The edge weights in the current sample were consistent with the bootstrapped sample, especially the connections with larger weights, indicating that the current network structure was stable among wave 1 and wave 2 (Supplementary Figure S2). The case-dropping subset bootstrap procedure showed that the betweenness, closeness, and strength values remained stable even after dropping large proportions of the sample (Figure 4). Compared with betweenness and closeness reported slightly low stability, the strength index in this sample was robust and trustworthy (i.e., CS-C = 0.75 in wave 1; CS-C = 0.67 in wave 1; i.e., after dropping up to 75% of the sample, the order of the symptoms in strength was still correlated with the original one (r = 0.7). Hence, we focused on the interpretation of symptom strength based on this network analysis.

**Figure 5:**
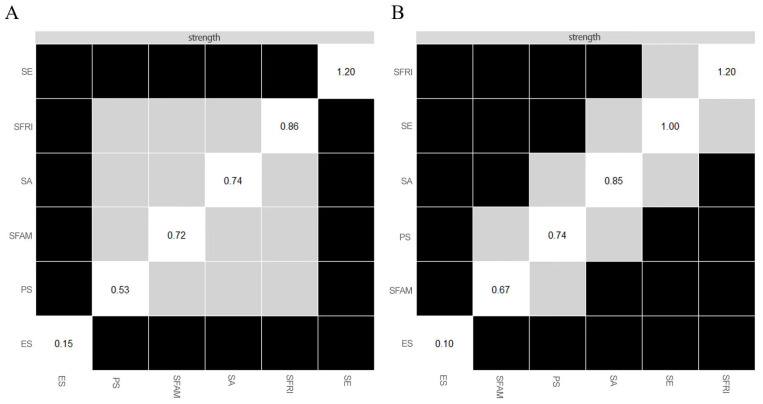
Estimation of node strength difference by bootstrapped difference test. Note, bootstrapped difference tests between node strength of factors. Grey boxes indicate nodes that do not significantly differ from one another. Black boxes represent nodes that differ significantly from one another (α = 0.05). White boxes show the values of node strength. (**A**), wave 1. (**B**), wave 2.

In terms of strength, node SE (i.e., self-efficacy) was statistically stronger compared to other symptoms in wave 1. Meanwhile, node SFRI (i.e., support from friends) was statistically stronger compared to other symptoms in wave 1 (Figure 5). The bootstrapped difference tests also revealed that a large proportion of the comparisons among the edge weights were statistically significant among wave 1 and wave 2 (Supplementary Figure S3).

### 3.4. Network Comparison across Two Waves

**Figure 6:**
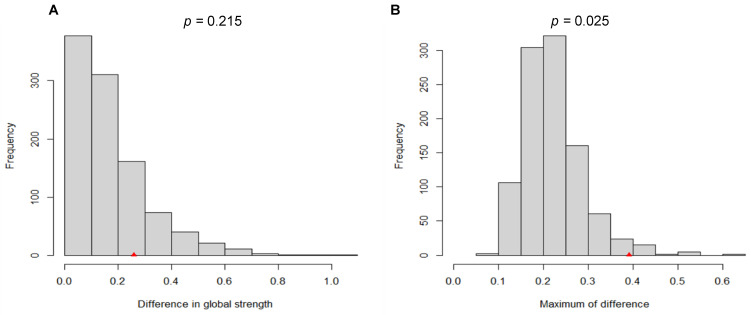
Comparison of network properties between wave 1 and wave 2 participants. Note (**A**) the plot of the bootstrap value of the difference in global network strength. (**B**) the plot of the bootstrap value of the maximum difference in any of the edge weights (1000 permutations).

The network comparison test (NCT) is a permutation test that is used to determine the invariance of different network features. As illustrated in Figure 6A, there was no significant difference in the bootstrap value of the global network strength (global network strength among wave 1 participants: 2.13; global network strength among wave 2 participants: 2.39; *S*: 0.26, *p* = 0.22). However, the bootstrap value for the largest difference in any edge weights (1000 permutations), as depicted in Figure 6B, was substantially different (*M* = 0.39, *p* = 0.03).

## 4. Discussion

To the best of our knowledge, there has been no prior research on network structures exploring the properties of resilience facets for university students via expressive arts therapy intervention. In sum, the present study has provided evidence supporting the effectiveness of expressive arts therapy in advancing resilience and changing the structure of resilience in the context of COVID-19. With the two-point tracking design, facets of self-efficacy, self-acceptance and problem-solving in resilience were notably improved following the intervention with the variable of emotional stability sustained at the mean level. Meanwhile, the network analysis has highlighted the central variable of self-efficacy in the pre-intervention and support from friends in the after-intervention. Furthermore, as determined by the NCT test, the network structure results were significantly different at two-wave points, and the connectivity among the three facets of problem solving, support from friends, and support from family was enhanced, with support from friends playing the role of hub nod in the following network. However, some results are worthy of discussion.

### 4.1. Facets of Self-Efficacy and Self-Acceptance

To begin with, the intervention of expressive arts therapy improved the resilience of university students on the mean level, especially significantly in self-efficacy, self-acceptance, which is consistent with previous studies [70]. As we know, self-efficacy was first introduced by Bandura and is often used interchangeably with self-confidence and the motivation to do well in practice [71]. In general, self-efficacy pertains to the individual’s confidence or belief in his or her abilities to accomplish a task or achieve a goal. The present participants have a comparatively low sense of efficacy before the intervention, which is in line with a previous Spanish study regarding the resilience of university students in the COVID-19 pandemic situation [16]. In accordance with the findings in this study, various therapeutic factors in arts therapy revealed the power in enhancing self-efficacy, such as the aesthetic experience in dance [72], and the self-awareness and motivation to change the way of life in psychodrama [73]. In other words, expressive arts therapy can greatly assist individuals in developing adequate self-confidence and trust to confront and resolve problems appropriately [74]. The supportive and affirmative environment that expressive arts interventions offered encouraged the clients to express emotional experience through expressive arts work, which in turn reinforced the self-efficacy of participants.

Additionally, individuals can easily develop self-acceptance because expressive arts therapy interventions are comfortable, joyful, harmonic, and creative [75]. Moreover, self-acceptance is the capacity to accept oneself without changing anything about one’s identity [76]. It is a healthy way of life that enables us to be ourselves without having to conceal aspects of our personalities or mask our emotions in front of others. Proper self-acceptance spurs individuals to develop an appreciation and love for their distinctive characteristics and those of others and tend to result in a good self-image and an increased sense of personal worth [77]. The nonjudgmental attitude employed through the workshop induced individuals to concentrate on the present reality, which in turn reduced the internalizing and externalizing behaviors in face of pressure [78]. Furthermore, the art therapy applied in this workshop reinforced the students’ ability in expressing complex feelings and sensations without verbal interpretations, which has been shown to be effective during the COVID-19 confinement [46]. A study using short-term group music therapy found distinctive efficacy in the promotion of students’ self-awareness and empathy as well [79].

One point that is worth being mentioned is that the pre-post networks were quite similar with respect to one feature and the interplay between self-efficacy and self-acceptance was markedly stable over the intervention period, that is, the strong association of these two variables held very consistently across the two waves, indicating that the role of self-efficacy and self-acceptance attributed the same importance to resilience. As the intrapersonal components of the resilience process, self-efficacy usually is developed in parallel to self-acceptance. Students with the ability to reevaluate experiences with a positive perception tended to accept the realities and struggle to work hard, even regarding failure as a valuable learning experience [40]. In addition, self-efficacy and self-acceptance were reported to be significant internal assets among protective factors in a COVID-related Canadian study sampled with ten Chinese international students by means of hermeneutic phenomenological inquiry [39]. The students believed in themselves to stay protected through the challenging times by use of spiritual and emotional control. Therefore, with the significant raise in the mean values considered, the impact in enhancing self-efficacy and self-acceptance by use of expressive arts therapy was quite prominent, and professional counselors should guide students to be aware of their positive strengths rather than clinically heal the trauma and stress.

### 4.2. Facets of Problem Solving, Support from Friends, and Support from Family

On the mean level, this study also found significantly increased competence in problem solving after the intervention. Given that resilience can be viewed as the capability to cope with stress, the coping strategy of problem solving is crucial in the subsystem of resilience. Effective problem solving is a necessity for maintaining a healthy mental state. The process of identifying and resolving problems is called problem-solving [80]. In order to solve a problem, the process of recognizing and assessing the situation, comprehending the underlying cause of the problem, determining the most feasible alternatives, establishing an action plan, and executing the plan [81] are important. By means of encouragement in expression, discussion, and change in perception, expressive arts therapy assisted individuals in identifying, analyzing, and resolving difficulties. Thus, the findings of this study confirm that expressive arts therapy is likely to considerably increase problem-solving abilities, which benefit individuals in shaping their coping mechanisms with potentially dangerous events and stress [82]. This is in line with the findings of a US study, which designed an eight-week expressive arts group curriculum to help first-year students cope with college demands [31]. Significant reductions in behavior problems have been reported through group activity counseling [31], and even the abilities in coping with the disturbance of addictive behaviors could be enhanced by consciously perceiving emotional distress, rebuilding a positive lifestyle, and raising self-efficacy of urge management in a short-term expressive arts therapy program [41]. The therapeutic change in arts therapy took place in the process of community building and collaborative working, with art as a medium for self-expression, creativity, and self-exploration. Through a whole range of expressive behaviors, individuals shaped their willingness to change their coping patterns. In addition, the group setting provided a safe environment for students to externalize their internal concerns and to bridge group dynamics and peer support.

Furthermore, the association of variables should be discussed through the differences in the linking patterns between the nodes within the two waves. From the perspective of network analysis, the study also confirmed that the architecture of resilience can be intervened by use of expressive arts therapy. Taking the strength of the node into consideration, the node of self-efficacy, followed by the node of support from friends, was the most connected variable with the others, indicating that it could play the most important role in resilience in the pre-intervention network. However, the magnitude of the node was converted after the intervention, with support from friends emerging as the greatest strength, followed by the node of self-efficacy.

The distinct shift of the central variable and the changed linking structure suggested that different coping mechanisms were applied. As far as wave 1 is concerned, problem solving primarily interacted with self-efficacy more strongly, which is in line with the pre-pandemic study of Spanish undergraduate students, showing that the coping strategies of problem-solving and self-efficacy were positively correlated and problem-solving served as a predictor of general perceived self-efficacy [83]. Likewise, problem solving in COVID-19 coping had positive associations with resilience and was predicted 24% of the total variance of the resilience, which was composed of personal competence and acceptance of oneself and life [16].

The network inference in wave 2 confirmed that support from friends represented the strongest node, and the variables of problem solving, support from friends, and support from family shaped a tight cluster. This suggested that the expressive arts therapy exerted an influence on the coping mechanisms of problem solving. The training in the form of music, dance, and drama particularly increased interaction and promoted social connection and cooperation, paired with art therapy, which stimulated verbalization, expression, and emotional awareness. After the intervention, problem solving appeared to be more distal with self-efficacy, but highly connect to support from friends and support from family. This finding clearly demonstrated that problem solving and social support jointly impacted the promotion of resilience after the therapy. The ability to link social network with problem solving was bolstered. Chinese students tended to utilize the strength of cohesion, and this sentiment was echoed by a qualitative inquiries research during the COVID-19 pandemic, stating that parental support and peer support appeared to be external resources of protective factors regarding resilience [39]. The affective and financial support from families and peers built an adaptive capacity in solving the current adversity, and the coping strategy of problem solving along with the external resources from parents and friends empowered the students to buffer against the negative effects in the face of the stress.

Before the intervention, problem solving that was based only on self-efficacy may limit one’s ability to handle difficulties successfully, while more access to friends and family would magnify the supportive effect of the peer network. The competency-based model of EuroPsy emphasized the importance of interpersonal skills and socioemotional skills with response to the capacity for resilience [16]. Based on communication theory of resilience, maintaining and using a communication network, which is a way to consolidate intimate relationships and expand one’s network, is one of the five major resilience processes that need to be enacted. When students faced financial challenges and disappointment due to the disruptive campus events caused by the pandemic, the students who garnered support by creating and leveraging relationship from friends and professors displayed more resilience to upset [40].

After the intervention, taking into consideration the dominant strength of support from friends in the coping mechanism, the influence of problem solving in resilience was primarily exerted through support from friends. The role of support from friends was central for the activation of the coping mechanism. This finding reinforced the significance of a peer-supportive system in expressive arts therapy in monitoring and promoting coping the strategy of problem solving in times of pandemic crisis. Students reaped the benefits of the positive cognitive effects by incorporating family and peer relationships into their lives, which urged the adoption of effective coping in problem solving. Consequently, the training in developing social resources for students under psychological disruptions should be highlighted, and peer-to-peer programs should be of great use to strengthen the coping mechanisms of college students during the pandemic.

### 4.3. Facet of Emotional Stability

As presented in the findings, the mean level of emotional stability did not change significantly. This could also be detected from the network structure, in which the variable of emotional stability appeared to be one distal facet, loosely connected with other components of resilience. Considering the two waves together, emotion stability remained the most stable variable, and it is worth mentioning the conclusion that expressive arts therapy could not impact emotion stability in this study. This is inconsistent with previous research using cartoon-based art therapy on high school students [84] and integrative therapy for children [85], which justified the efficacy in the advance of emotion stability. This result may be interpreted by the theory of personality trait change. A systematic review of 207 studies regarding the clinical intervention of personality traits concluded with the following three interesting findings: marked changes in personality traits were associated with interventions over an average time of 24 weeks; the extent to which personality traits changed had a loose relationship with the type of therapy; patients with anxiety disorder displayed the most change and patients with substance use changed the least [86]. Hence, it is reasonable to presume that emotional stability, as one of the big five trait dimensions [87], cannot enact the changing processes easily over a short period of time, especially for nonclinical people, whatever measures of therapy are used. To probe into the effect of a more significant change in emotion stability, a future study may conduct comparatively long-term therapy in a clinical diagnosed sample with anxiety disorder.

Limitations of the present research should be acknowledged. First, as a pilot study using network analysis, this study required data composed of comparatively large samples. In addition, the long-term psychological pressure of Chinese college students has been proved by the longitudinal studies and cannot be self-healed spontaneously. Therefore, this intervention did not apply an experimental pretest-posttest control group design due to limited sample resource, which restricted the interpretability of the results. To enhance the effectiveness of intervention, a control intervention is suggested in future studies. Second, as a nonclinical intervention, although this study emphasized that participants had recently experienced negative emotional distress brought by negative events while recruiting participants, it did not categorize them through clinical diagnoses, raising doubts about whether the expressive arts therapy intervention is beneficial for unique psychiatric problems. Hence, future studies may involve more stratified and targeted recruitment to enhance the potential generalization. Third, no significant variation in emotional stability was found via the treatment in this study, and future studies could further elucidate this crucial aspect of resilience with appropriate therapy designs and more sensitive measures of expressive arts therapy. Fourth, while the study employed a longitudinal intervention of four sessions to assess the effect of expressive arts therapy on improving individual resilience, a longer period of intervention and follow-up measurement could be conducted to detect the continuous changes in resilience in the network. Finally, the cross-validation of the evaluation of the intervention should rely on the replication in other samples, derived from the same population of university students in a diverse cultural context.

## 5. Conclusions

This study for the first time evaluated and confirmed the validity of the impact of expressive arts therapy in enhancing resilience for university students by adopting network analysis. In conclusion, the present study deciphered the relationship between the six components of resilience (i.e., self-efficacy, self-acceptance, emotional stability, problem-solving, support from friends and support from family) before and after intervention. On the mean level, self-efficacy, self-acceptance and problem-solving improved significantly. From the perspective of network analysis, self-efficacy emerged as the strongest node of the resilience network among wave 1, while support from friends was the most central node among wave 2. The change in core symptoms demonstrated the effectiveness of the therapeutic expressive arts intervention in detecting significance in the interactions between problem solving, support from friends and support from family. This treatment in our study stressed that the capability of problem solving was important to social support, especially in the support from friends. It is worth mentioning that problem solving appeared to shift connections from self-efficacy before the treatment to support from friends and support from family after the intervention. Meanwhile, self-efficacy and self-acceptance were highly connected with the rest of the network, both among the two waves, suggesting that self-efficacy and self-acceptance played significant roles in the interplay of resilience.

Taken together, these findings advance the applications of expressive arts therapy in the enhancement of resilience under the COVID-19 context and pave the way for the network analysis approach in the evaluation of expressive arts therapy for future studies. In order to improve the capacities in problem solving for university students in the worldwide pandemic, the targets of intervention should be calibrated to the cultivation of social support networks, especially in the support from friends. Expressive arts therapy has provided a remedy for the mental health intervention of university students.

## Figures and Tables

**Table 1:** The descriptive statistic and paired *t*-test among wave 1 and wave 2.

Variable	Wave 1 (Mean ± SD)	Wave 2 (Mean ± SD)	*p*	Cohen’d
SE	3.72 (0.71)	3.96 (0.71)	<0.001	0.34
SA	3.60 (0.87)	3.94 (0.77)	<0.001	0.42
ES	3.18 (0.89)	3.12 (0.86)	0.431	0.07
PS	3.83 (0.70)	4.02 (0.70)	0.002	0.28
SFRI	4.04 (0.64)	4.11 (0.68)	0.24	0.10
SFAM	3.99 (0.81)	4.08 (0.78)	0.207	0.11

Note. SE, self-efficacy. SA, self-acceptance. ES, emotional stability. PS, problem solving. SFRI, support from friends. SFAM, support from family.

## Data Availability

The data are available from the corresponding author upon reasonable request (may require data use agreements to be developed).

## Supplementary Materials

The following supporting information can be downloaded at: https://www.mdpi.com/article/10.3390/ijerph19137658/s1, Table S1: The descriptive statistic of wave 1 datasets; Table S2: The descriptive statistic of wave 2 datasets; Table S3: The weighted matrix among wave 1; Table S4: The weighted matrix among wave 2; Figure S1: Network model for the six variables of resilience among wave 1 and wave 2; Figure S2: Bootstrapped confidence intervals of edge weights; Figure S3: Estimation of edge weight difference by bootstrapped difference test.
