# Supplementary material for: Evaluation of Expressive Arts Therapy on the Resilience of University Students in COVID-19: A Network Analysis Approach
Source: Int J Environ Res Public Health. 2022 Jun 23;19(13):7658. doi: 10.3390/ijerph19137658 (PMC9265567; doi:10.3390/ijerph19137658)
Supplement: Supplementary file 1 [file ijerph-19-07658-s001.zip › ijerph-1721765-supplementary.pdf]

Table S1. The descriptive statistic of wave 1 datasets.

Variable	*mean*	*sd*	*median*	*min*	*max*	*range*	*skew*	*kurtosis*	*se*
SE	3.72	0.71	3.80	1.00	5.00	4.00	-0.38	1.15	0.04
SA	3.60	0.87	3.67	1.00	5.00	4.00	-0.54	0.54	0.05
ES	3.18	0.89	3.14	1.00	5.00	4.00	0.04	-0.65	0.05
PS	3.83	0.70	4.00	1.00	5.00	4.00	-0.36	0.64	0.04
SFRI	4.04	0.64	4.00	1.86	5.00	3.14	-0.45	-0.13	0.04
SFAM	3.99	0.81	4.00	1.00	5.00	4.00	-0.74	0.77	0.05

Note. SE, Self Efficacy. SA, Self Acceptance. ES, Emotional Stability. PS, Problem Solving. SFRI, Support From Friends. SFAM, Support From Family.

Table S2. The descriptive statistic of wave 2 datasets.

Variable	*mean*	*sd*	*median*	*min*	*max*	*range*	*skew*	*kurtosis*	*se*
SE	3.96	0.71	4.00	1.00	5.00	4.00	-0.44	0.72	0.04
SA	3.94	0.77	4.00	1.00	5.00	4.00	-0.73	0.90	0.05
ES	3.12	0.86	3.00	1.43	5.00	3.57	0.09	-0.62	0.05
PS	4.02	0.70	4.00	1.00	5.00	4.00	-0.64	1.44	0.04
SFRI	4.11	0.68	4.14	1.57	5.00	3.43	-0.45	-0.23	0.04
SFAM	4.08	0.78	4.00	1.00	5.00	4.00	-0.68	0.39	0.05

Note. SE, Self Efficacy. SA, Self Acceptance. ES, Emotional Stability. PS, Problem Solving. SFRI, Support From Friends. SFAM, Support From Family.

Table S3. The weighted matrix among wave 1.

	SE	SA	ES	PS	SFRI	SFAM
SE	0	0.745	0	0.282	0.132	0.091
SA	0.745	0	0	0	0	0
ES	0	0	0	0	0	0.154
PS	0.282	0	0	0	0.252	0
SFRI	0.132	0	0	0.252	0	0.473
SFAM	0.091	0	0.154	0	0.473	0

Note. SE, Self Efficacy. SA, Self Acceptance. ES, Emotional Stability. PS, Problem Solving. SFRI, Support From Friends. SFAM, Support From Family.

Table S4. The weighted matrix among wave 2.

	SE	SA	ES	PS	SFRI	SFAM
SE	0	0.742	0	0.073	0.046	0.148
SA	0.742	0	0	0.112	0	0
ES	0	0	0	0	0.102	0
PS	0.073	0.112	0	0	0.560	0
SFRI	0.046	0	0.102	0.560	0	0.519

SFAM	0.148	0	0	0	0.519	0

Note. SE, Self Efficacy. SA, Self Acceptance. ES, Emotional Stability. PS, Problem Solving. SFRI, Support From Friends. SFAM, Support From Family.

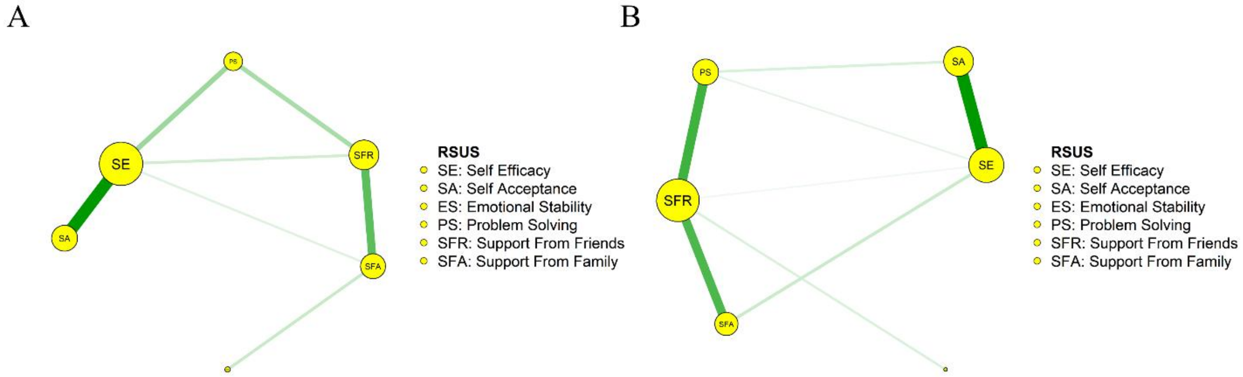

Figure S1. Network model for the six variables of resilience among wave 1 and wave 2.

Note. Positively worded items were reversed prior to the network estimation. A, wave 1. B, wave 2. Green line indicates positive correlation. The size of the node represents the original value of the node strength.

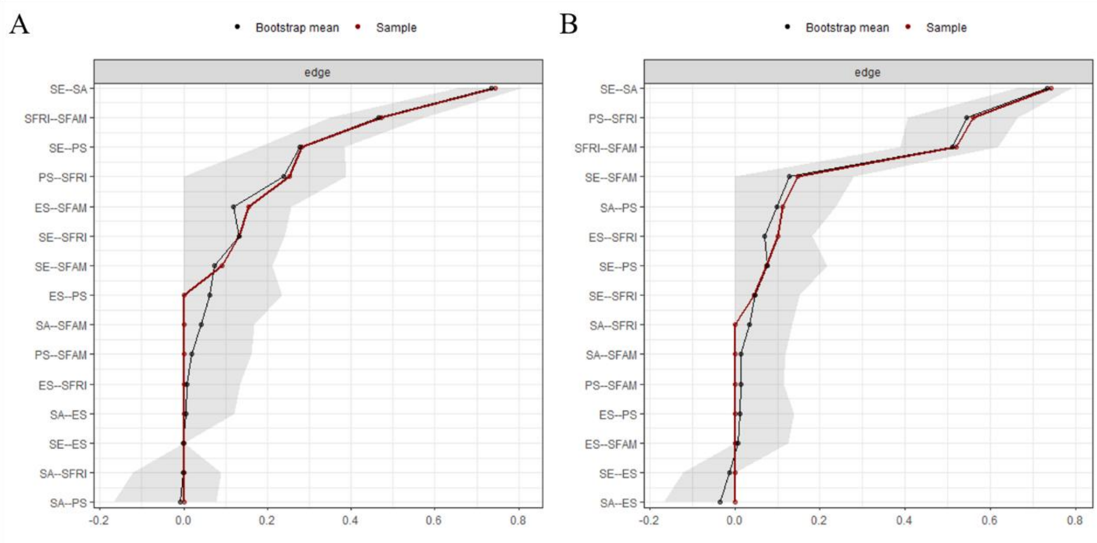

Figure S2. Bootstrapped confidence intervals of edge weights.

Note. A, wave1. B, wave2.

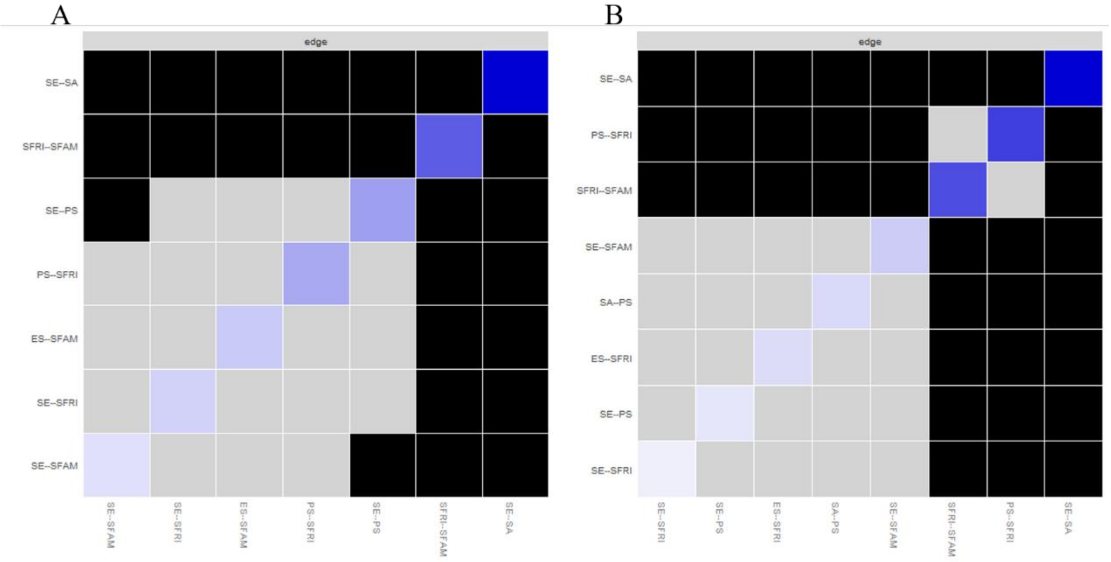

Figure S3. Estimation of edge weight difference by bootstrapped difference test.

Note. Bootstrapped difference tests between edge weights in the network. Grey boxes indicate edges that do not significantly differ from one another. Black boxes represent edges with a significant difference from one another (α = 0.05). Blue boxes in the edge-weight plot indicate positive correlations. A, wave1. B, wave2.
